# A case report of a patient with metastatic ocular melanoma who experienced a response to treatment with the BRAF inhibitor vemurafenib

**DOI:** 10.1186/s12885-016-2657-7

**Published:** 2016-08-12

**Authors:** A. Maleka, G. Åström, P. Byström, G. J. Ullenhag

**Affiliations:** 1 Department of Oncology, Uppsala University Hospital, 751 85 Uppsala, Sweden; 2 Department of Immunology, Genetics and Pathology, Uppsala University, Uppsala, Sweden; 3 Department of Surgery, Section of Radiology, Uppsala University, Uppsala, Sweden; 4 Novartis Sverige AB, Kemistvägen 1, 183 79 Täby, Sweden

**Keywords:** BRAF inhibitor, BRAF mutation, Conjunctival malignant melanoma, Ocular melanoma, Vemurafenib

## Abstract

**Background:**

Conjunctival malignant melanoma (CMM) is a rare malignancy and in the advanced setting there is no effective treatment. In contrast, half of cutaneous melanomas have BRAF mutations and treatment with BRAF inhibitors is established for patients with disseminated disease. The most common form of ocular melanoma, uveal melanoma, lacks these mutations, however, their presence has been reported for CMM.

**Case presentation:**

We used the BRAF inhibitor vemurafenib to treat a 53 year-old female suffering from a BRAF^V600E^ mutated metastatic CMM. The patient benefited from the treatment, a response was evident within a week and she experienced a progression free survival of four months.

**Conclusions:**

To our knowledge, this is the first described case of response to vemurafenib treatment in a patient with ocular melanoma.

## Background

Two subtypes of primary ocular melanoma have been described, uveal and conjunctival. Conjunctival malignant melanoma (CMM) is a rare condition with an incidence of 0.2 to 0.8 per million in Caucasian populations. It is a frequently lethal non-cutaneous neoplasm with an average 10-year mortality rate of 30 % [1]. Studies over the past two decades have revealed different genetic subsets of melanoma [2–4]. Half of cutaneous melanomas harbor activating mutations in BRAF and the most abundant is BRAF^V600E^ followed by BRAF^V600K^. However, the most common form of ocular melanoma, uveal melanoma, lacks these mutations except from its smallest subgroup, iris melanoma. CMMs have not been well characterized at the genetic level, however, BRAF^V600E^ mutations have been reported in 14 % to 50 % [5–7].

At present no effective treatment is available for metastatic CMM, hence the need for new therapies is essential. In contrast, treatment with the BRAF inhibitors vemurafenib and dabrafenib is established for patients with BRAF^V600E^ and BRAF^V600K^ mutated disseminated cutaneous melanomas [8, 9]. BRAF status might also be a predictive marker in deciding whether to use BRAF inhibitors for the treatment of patients with advanced CMM [10]. Here we present a case of a patient with metastatic CMM positive for the BRAF^V600E^ mutation who was treated with vemurafenib. To our knowledge, there is no previously described treatment response to vemurafenib in ocular melanoma.

## Case presentation

**Fig. 1:**
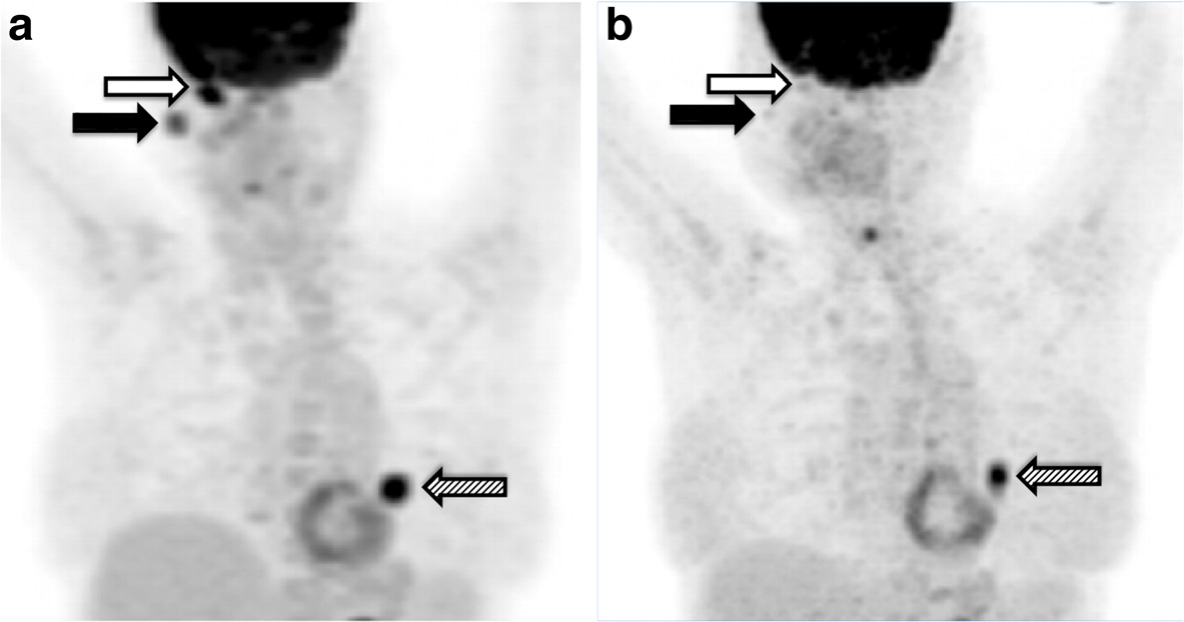
**a** FDG-PET/CT in December 2012, prior to BRAF inhibitor therapy, showed intensely FDG avid lesions in the orbit, the parotid glad and the lung post AdCD40L treatment. **b** PET/CT in May 2013, after the initiation of the treatment with vemurafenib, showed that all the previously described intensely FDG avid lesions had lower SUVmax uptake. Physiologic FDG uptake in right posterior vocal cord is observed. Black arrow: metastasis in right parotid gland. White arrow: metastasis in right orbit. Striped arrow: lung metastasis

The patient, a 53-year old Caucasian woman, initially noticed a lesion in her right eye. After a medical appointment at the ophthalmologic clinic at a regional hospital, a decision to remove the lesion was taken and an operation was carried out in August 2011. The pathology report showed a 13×11×7 mm malignant melanoma located in the conjunctiva with a minimal resection’s margin. The patient was re-operated one month later and the pathology report revealed a remnant of the melanoma with still a minimal resection’s margin. Therefore, the patient received cryotherapy. Four months after the first surgical procedure five new tumor lesions were detected in the same eye. Treatment with mitomycin eye drops was initiated, however enucleation of the right eye had to be carried out two months later to obtain local control. One month post enucleation, a CT scan of the chest and abdomen showed no metastases. However, yet two months later, positron emission tomography with 2-deoxy-2-[fluorine-18] fluoro-D-glucose integrated with computed tomography (18 F-FDG PET/CT also referred as PET/CT scan) revealed an orbital, a parotid gland and a suspected lung metastasis. Treatment with temozolomide was started and carried on for five months until progressive disease in all locations including the lung was noted in a new PET/CT scan. Shortly thereafter, the patient was included in a trial and received immunostimulatory gene therapy with the investigational drug AdCD40L in combination with low dose cyclophosphamide. Specifically, the patient received four weekly ultrasound-guided intratumoral injections in the parotid gland. Three days after the final injection, a CT-brain scan was performed due to left-sided leg weakness and revealed bleeding brain metastases. An MRI scan confirmed the presence of five brain metastases and the patient received whole brain radiotherapy (4 Gy × 5). A PET/CT scan at that time point showed progression in all lesions except the parotid compared with the most recent PET/CT scan (Fig. 1a).

Tissue from the primary tumor was tested for BRAF status, revealing the presence of the V600E mutation.

**Fig. 2:**
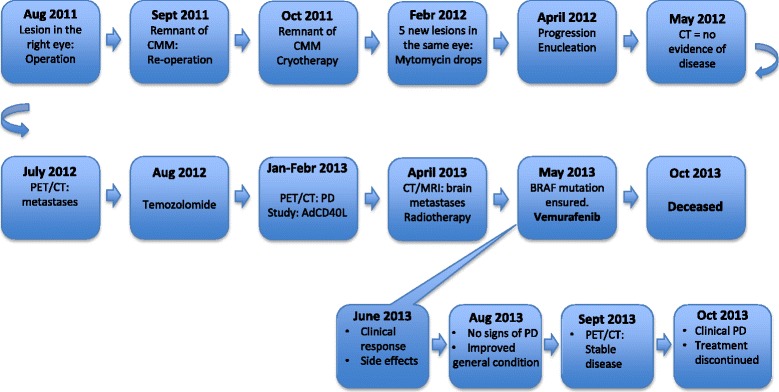
Schematic timeline from the day the patient was diagnosed with conjunctival malignant melanoma (CMM) until she was deceased. PD: progressive disease

One month after the whole brain radiotherapy, vemurafenib treatment (standard dose: 960 mg po q 12 h) was initiated. The patient responded rapidly to the treatment; the metastases in the parotid gland and orbit were reduced in size within a week. After two weeks of therapy, the patient experienced maculopapular rash located on the head’s uppermost part, classified as grade 2 according to common terminology criteria for adverse events (CTCAE version 4.0), and the treatment was paused. One week later the rash was improved to grade 1 and the treatment was re-started at a lower dose (25 % reduction of the initial dose), whereafter no side effects were observed. After four weeks of vemurafenib treatment, the above-described clinically detectable metastases in the parotid gland and orbit had disappeared. At the next clinical examination, yet two months later, the patient’s general condition was considerably improved without clinical signs of disease progression. A PET/CT scan one month later, confirmed the reduction of tumor burden in all locations compared to the pretreatment examination (Fig. 1b). In particular, the parotid metastasis had only minor residual FDG-uptake and the size of and the FDG-uptake in the lung metastasis had decreased. However, at that time the orbital lesion had clinically reoccurred, measuring one centimeter in diameter indicating progressive disease. Based on this latter finding in combination with the worsened general condition of the patient, the treatment was assessed as no longer effective and was discontinued. The total duration of the BRAF therapy was four months. A new CT scan of the brain was planned in order to map the brain metastases and determine the possibility to repeat radiotherapy. However, the patient’s clinical condition quickly deteriorated. Therefore, she underwent the CT scan earlier than scheduled whereby more brain metastases, than had previously been detected, were diagnosed. The patient passed away the day after. The time schedule for the case is depicted in Fig. 2. The pathology report was eventually reviewed and it was ensured that the diagnosis indeed was CMM.

## Discussion

We report on a patient with disseminated CMM who was treated with the BRAF kinase inhibitor vemurafenib due to the presence of the BRAF^V600E^ mutation. She had received all established treatments and even experimental therapy; AdCD40L.

Ocular melanoma is a rare type of malignant melanoma. For most small and medium size tumors, irradiation is recommended. Surgery is often the treatment of choice for recurrent disease, after initial radiotherapy. Approximately half of the patients with ocular melanoma develop metastatic disease [11]. In general, prognosis is very poor for patients with advanced disease, and without treatment the median survival is around eight months [12].

In the common clinical practice, all patients with advanced cutaneous malignant melanoma who meet the criteria for treatment with a BRAF inhibitor are tested for the presence of the BRAF mutation [13]. However, BRAF therapy is not established for patients with CMM or other ocular melanomas. Vemurafenib is a highly selective inhibitor of mutated BRAF, it induces objective responses in 50 % of patients and prolongs survival when compared to traditional chemotherapeutic agents [14–16]. The drug is even effective in patients with brain metastases [17]. Unfortunately, most, if not all, patients eventually develop resistance to vemurafenib [18–20]. For many years it was thought that patients with ocular melanoma could not benefit from treatment with BRAF-kinase inhibitors due to the fact that the RAS-BRAF kinase pathway is not involved in the most common ocular melanoma, the choroidal melanoma [10, 21]. However, it was eventually shown that BRAF mutations are present in conjunctival melanomas [5, 7].

Since the patient had received and experienced disease progression on all established treatments, vemurafenib treatment was considered an option. A clear correlation between the on-set of vemurafenib therapy and the regression of the metastases in the orbit and parotid gland was clinically observed. However, it cannot be ruled out that the major regression of the metastasis of the orbit partly was a result of the whole brain radiotherapy. It is also unclear whether the response in the parotid gland represents an effect of vemurafenib alone. A late synergistic effect with gene therapy (AdCD40L) is possible despite the obvious systemic resistance to that treatment as pointed out with the occurrence of brain metastases after the last injection of AdCD40L. Of note is that there was a clear response in the non-localized treated lung metastasis emphasizing that the vemurafenib treatment was beneficial. In the only conducted study with AdCD40L administered in metastatic melanoma patients no late immune responses were noted [22]. In addition, other immunotherapy approaches in ocular melanoma patients have not proved effective in contrast to cutaneous melanoma. In fact, treatment with the anti-CTLA-4 antibody ipilimumab showed limited treatment benefit [23–25] and preliminary data from ongoing clinical trials with PD-1 antibodies are not encouraging [26]. It is therefore unlikely that the patient’s response represents a late systemic synergistic effect with AdCD40L treatment.

The treatment was well tolerated after an early 25 % reduction of the initial dose. The patient’s general condition was considerably improved alongside with rapid regression of tumor lesions. The patient passed away five months after the initiation of the treatment with vemurafenib, shortly after the treatment’s discontinuation.

According to the registration trial and Drummer et al. [14–17] the median progression free survival after vemurafenib treatment is 3.9 months for patients with BRAF^V600E^-mutant metastatic cutaneous malignant melanoma with non-excisable previously treated brain metastases. The patient described in this case report clearly benefitted from the treatment and the gain was very similar to the average for the corresponding group of patients with cutaneous malignant melanoma.

It is reasonable to believe that all patients with BRAF-mutant cancer would benefit from treatment with BRAF inhibitors. However, colon cancer patients harboring the BRAF^V600E^ oncogenic lesion have a poor prognosis and do not respond to vemurafenib therapy. It was shown that this unresponsiveness depends on BRAF inhibition through feedback activation of EGFR [27].

Two attempts of treating metastatic CMM with vemurafenib have previously been reported. One of these patients experienced a mixed response, which after a short period was followed by evident disease progression [28]. In a Chinese CMM trial one of the patients’ tumor was tested positive for the BRAF mutation and treatment with vemurafenib was given. However, the outcome was unclear for this second reported case [29]. In addition, a patient who received dabrafenib experienced an objective response but disease progression was evident after 6 months [30].

## Conclusions

In conclusion, we show for the first time that treatment of BRAF mutated metastatic CMMs with vemurafenib could be of value. Further studies are needed to assess the efficacy of BRAF and PD1 inhibitors in the different subtypes of ocular melanoma.

The CMM subtype of ocular melanoma is however very rare making it extremely difficult to perform a randomized clinical study.
